# Pharmacological potential of the histone demethylase inhibitor GSK-J4 in mitigating NF-κB-mediated inflammatory cascades associated with autoimmune disorders

**DOI:** 10.1007/s10787-026-02223-4

**Published:** 2026-04-20

**Authors:** Ghada Nour Eldeen, Mona F. Sokkar, Randa S. Lotfy, Hala M. Raslan, Manal Abd El Moniem El Menyawi, Kamal A. El-Atrebi, Hala T. El Bassyouni

**Affiliations:** 1 Molecular Genetics and Enzymology Department, Human Genetics and Genome Research Institute, National Research Centre (NRC), Cairo, Egypt; 2 Stem Cell Research Group, Medical Research Centre of Excellence, National Research Centre (NRC), Cairo, Egypt; 3 Internal Medicine Department, Medical Research and Clinical Studies Institute, National Research Centre (NRC), Cairo, Egypt; 4 Rheumatology and Clinical Immunology Unit, Internal Medicine Department, Faculty of Medicine Kasr Alainy, Cairo University, Cairo, Egypt; 5 General Medicine and Hepatology Department, National Hepatology and Tropical Medicine Research Institute, Cairo, Egypt; 6 Clinical Genetics Department, Human Genetics and Genome Research Institute, National Research Centre (NRC), Cairo, Egypt

**Keywords:** Autoimmune diseases, Inflammasome, JMJD3, NF-κB, Cytokines, GSK-J4

## Abstract

Inflammasome-related sterile inflammation is a leading cause of several autoimmune and rheumatic disorders, including rheumatoid arthritis (RA), systemic lupus erythematosus (SLE), and inflammatory bowel disease (IBD). Although the exact causes of rheumatic diseases remain unclear, it is evident that ongoing inflammatory reactions are the main factors driving their development. Jumonji domain-containing protein-3 (JMJD3) is an epigenetic modifier that can strongly promote the inflammatory process in many diseases by impacting the Nuclear Factor-κB (NF-κB) signaling. Meanwhile, GSK-J4 has been identified as a selective inhibitor of JMJD3. Therefore, our study aimed at exploring the effects of JMJD3 inhibition by GSK-J4 on inflammasome activation and oxidative stress release in lipopolysaccharide (LPS)-primed patient-derived adherent mononuclear cells of different autoimmune diseases: RA, SLE, and IBD. The inflammatory response was evaluated prior to and following the GSK-J4-cell treatment by assessment of gene and protein expression of inflammatory factors using qRT-PCR and Western blot, respectively. Additionally, cytokine and nitric oxide release in culture supernatants was measured using enzyme-linked immunosorbent assay (ELISA). Our results showed a significant correlation between the inhibition of JMJD3 and the attenuation of inflammatory signaling pathways through reduced NF-κB activation, diminished expression of pro-inflammatory cytokines, and decreased inflammatory stress response. These findings supported the idea that epigenetic modification is a crucial mechanism for priming the inflammasome activation in various autoimmune diseases, confirming the potential of epigenetic factors as prospective therapeutic targets for immune-mediated inflammatory diseases.

## Introduction

Chronic inflammation in autoimmune disorders is associated with innate immunity activation, epigenetics, and transcriptional control of inflammatory responses (Xiang et al. 2023; Guo et al. 2024). Among the complex processes that regulate chronic inflammation, NF-κB-mediated inflammatory priming and inflammasome stimulation, have attracted much attention in controlling pathological immune responses (Liu et al. 2017).

One of the essential components of the inflammatory network is the inflammasome, which is a cytosolic complex of several proteins playing an essential role in activating inflammatory responses that is activated through two consecutive steps. First, NF-κB priming activates NLRP3, pro-IL-1β, and pro-IL-18 with the association of the inflammasome. Second, the activation of caspase-1 following the inflammasome formation through signals of cellular stress. Therefore, abnormal activation of the inflammasome is the fundamental mechanism through which immune complex-driven inflammation occurs in SLE, mucosal immune activity occurs in IBD, and joint tissue damage occurs in RA (Guo et al. 2020). Therefore, sustained NF-κB activation is critical for the activation of autoimmune diseases by enhancing the inflammatory response. Recent studies revealed the contribution of epigenetic regulators particularly a histone demethylase, the Jumonji domain-containing protein-3 (JMJD3), as a key player in the regulation of inflammatory gene expression.

Epigenetic control acts as a switch for inflammatory genes, especially during macrophage differentiation. This epigenetic regulation involves histone modifications, usually at lysine residues, which often get methylated and acetylated. Histone H3 lysine 27 trimethylation (H3K27me3) modulates gene transcription, and it is essential for tissue homeostasis during inflammatory responses. The H3K27 residue is usually modified by the methylation carried out by Enhancer of Zeste Homolog 2 (EZH2) that can be reversed by JMJD3 and Ubiquitously Transcribed Tetratricopeptide Repeat on Chromosome X (UTX) (Zhang et al. 2018).

Jumonji domain-containing protein D3 (JMJD3), also called lysine-specific demethylase 6B (KDM6B), is a member of the JMJC histone demethylase family that regulates the expression of the associated genes by selectively demethylating trimethylated H3 lysine 27 (H3K27me3) (Ding et al. 2021). JMJD3 is a conventionally “repressive” histone modification, and numerous investigations have revealed its role in epigenetic control of genes that promote the inflammatory reactions (Zhang et al. 2019). Additionally, a prior investigation demonstrated the potential interaction of JMJD3 and NF-κB in several inflammatory diseases, including those mediated by lipopolysaccharide (LPS) (Yu et al. 2017). Thus, targeting the activity of JMJD3 with small molecule agents has appeared as an interesting therapeutic strategy for the mitigation of inflammatory reactions. Huang et al. (2020) and Leng et al. (2022) have mentioned that dysregulated activity of JMJD3 could contribute to autoimmune disorders by enhancing inflammatory gene transcription.

GSK-J4, a cell-permeable inhibitor of JMJD3 and its closely related family member UTX, has been shown to inhibit the removal of the repressive H3K27me3 marks and inhibit the transcriptional activity of NF-κB-dependent inflammatory gene expression (Kruidenier et al. 2012). GSK-J4 has also been demonstrated to exhibit a therapeutic potential by reducing cytokine production, macrophage activation, and disease outcome in experimental autoimmune/inflammatory diseases (Doñas et al. 2016). However, the extent to which the inflammatory pathways are influenced by JMJD3 inhibition in patient-derived immune cells with different autoimmune diseases is still not fully understood.

Although RA, SLE, and IBD display highly heterogeneous patterns of immune disruption, they appear to originate from the same inflammatory pathway of the activation of NF-κB and the inflammasome priming. Therefore, examining the role of the epigenetic regulators like JMJD3 in the same experimental model could reveal the general pathways underlying autoimmune inflammatory reactions. Thus, the current study intended to investigate the effect of the attenuation of the epigenetic regulator, JMJD3, by the inhibitor GSK-J4 in the context of the NF-κB-mediated inflammatory process. The model adopted was LPS-primed PBMC-derived monocytes from RA, SLE, and IBD patients. Furthermore, we aimed to provide more clinical relevance to the exploration of the shared mechanisms of the inflammatory regulation of these autoimmune diseases by using the immune cell responses of patients rather than using cell line-based approaches.

## Patients and methods

### Patients

The study included thirty cases: ten with rheumatoid arthritis (RA), ten with systemic lupus erythematosus (SLE), and ten individuals with inflammatory bowel Disease (IBD). Participants were recruited from the Clinical Genetic Clinic and the Rheumatology Clinic at the Medical Research Centre of Excellence, National Research Centre (NRC), Egypt. SLE participants were also referred from the Rheumatology Clinic at Kasr Al-Ainy Hospital, Egypt. The study was approved in the 6th of February, 2023, by the Medical Research Ethics Committee (MREC) of the NRC approval No. 13060165-1, in accordance with the ethical standards of the Declaration of Helsinki, 1975. Written informed consent was obtained from all participants following a clear explanation of the study’s objectives and procedures.

## Clinical and laboratory investigations

All participants underwent comprehensive history taking and a detailed clinical examination. Blood samples (10 ml) were drawn from all included cases for routine laboratory investigations and peripheral blood mononuclear cell (PBMC) isolation. All diagnoses were established according to internationally accepted classification criteria: the 2010 American College of Rheumatology/European League Against Rheumatism (ACR/EULAR) criteria for RA (Aletaha et al. 2010), the American College of Rheumatology (ACR) classification criteria for SLE (Aringer et al. 2019), and standard clinical, endoscopic, radiological, and histopathological criteria for IBD (Cicero and Mazziotti 2021).

The diagnosis of all enrolled participants was verified with proof of active illness at recruitment. Validated indices were used to assess disease activity, including the Disease Activity Score-28 (DAS28) for RA, Systemic Lupus Erythematosus Disease Activity Index (SLEDAI) for SLE, and clinical, endoscopic, and biomarker parameters such as fecal calprotectin and C-reactive protein for IBD. Exclusion criteria included acute or chronic infections, cancer, pregnancy, concurrent immunological disorders, recent vaccinations or intensive immunosuppressive therapies, biologic drugs, or high-dose corticosteroids in the last 4 weeks before sampling.

## Isolation and culture of peripheral blood mononuclear cells (PBMCs)

PBMCs from all cases were isolated by Ficoll-Hypaque density gradient centrifugation at room temperature (20 °C). Heparinized whole blood was layered onto an equal volume of phosphate buffer saline solution (PBS) (pH 7.2) in a conical centrifuge tube. Then, the same volume of Ficoll-Hypaque medium was taken. The suspension was centrifuged at 3500 rpm for 30 min at room temp. The PBMC layer was collected and then washed three times with PBS. The PBMCs were grown at 37 °C with 5% CO_z_ in DMEM medium (Gibco^®^ Invitrogen) supplemented with 10% heat-inactivated FBS (Gibco), 2 mmol/L L-glutamine, and 100 IU/ml penicillin and streptomycin (Gibco). After 3 days, the supernatant and the non-adherent cells were removed, and the adherent PBMCs were used for further experiments (Jonitz-Heincke et al. 2017). Following adherence selection, the remaining cells predominantly consisted of monocyte-derived adherent immune cells; therefore, these are referred to as adherent PBMCs.

## GSK-J4 treatment of LPS-primed monocytes

We set up a controlled experiment to understand the anti-inflammatory effects of GSK-J4. Hence, this experiment involved three groups: untreated control cells, LPS-stimulated cells, and LPS-stimulated / GSK-J4-treated cells. Adherent mononuclear cells from cases with RA, IBD, or SLE were plated at 2.5 × 10^4^ cells per well in 6-well plates. We used DMEM, GlutaMAX medium (Thermo Fisher Scientific), with 10% fetal bovine serum (Lonza), and let them culture overnight. The next day, we primed the target cell groups with 1 µg/mL lipopolysaccharide (LPS; Sigma-Aldrich) for 24 h (Zhao et al. 2022a) with further treatment with GSK-J4 (10 μm) for 24 h. This dose was chosen based on previous work indicating that it efficiently inhibits JMJD3-dependent H3K27 demethylation and proinflammatory cytokine expression without causing cytotoxicity. Furthermore, the CCK-8 assays were performed to detect cell viability for verifying that no cytotoxicity of the chosen concentration.

The effect of GSK-J4 was analyzed with ELISA for measuring the levels in inflammatory markers such as IL-6 and IL-1β, and at the gene and protein expression level by quantitative PCR and Western blot respectively (Lu et al. 2023), allowing us to distinguish between the baseline inflammation induced by LPS and the epigenetic modifications induced by GSK-J4 treatment (Kruidenier et al. 2012; Doñas et al. 2016).

## CCK-8 proliferation assay

Following the manufacturer’s instructions, the CCK 8 test was used to evaluate cell viability. Briefly, adherent mononuclear cells were plated into 96-well culture plates at a density of 5 × 10³ cells per well in Dulbecco’s Modified Eagle Medium (DMEM) supplemented with complete nutrients. The cells were incubated overnight at 37 °C in a humidified incubator with 5% CO₂. Three groups of cells were evaluated: LPS-primed, LPS-primed/GSK-J4-treated, and the normal control group (non-primed and non-treated). Following the designated incubation period with LPS and LPS/GSK‑J4, 10 µL of Cell Counting Kit‑8 (CCK‑8) reagent was added to each well, and the plates were incubated for an additional 1 h. At a wavelength of 450 nm, the optical density (OD) was then measured. The following formula was used to calculate the proportion of viable cells: OD (treated) - OD (blank) / OD (control) OD (blank) = x100 = ratio (%) (Xu et al. 2018).

### Relative gene expression analysis

The Direct-zol RNA Miniprep Kit (ZYMO, USA) was used to isolate total RNA from different cell groups. Using the COSMO cDNA synthesis kit (Willowfort, UK), reverse transcription was accomplished. qRT-PCR assays were performed on the LightCycler^®^ 480 real-time PCR system (Roche, Germany) utilizing the HERA SYBR^®^ Green qPCR Kit (Willowfort, UK). Glyceraldehyde-3-phosphate dehydrogenase (GAPDH) was used as a reference gene. CT, ΔCT, and ΔΔCT were then calculated, and relative expression of the housekeeping and target genes was quantified using the following equation: relative quantification (RQ) = 2^−ΔΔCT^.

The list of the studied genes and the sequences of primers used for gene expression analysis are illustrated in Table 1.

**Table 1:** Sequences of primers for qRT-PCR assay

Gene	Forward primer	Reverse primer
NF-κB p65	5′-GCAGCACTACTTCTTGACCACC-3′	5′-TCTGCTCCTGAGCATTGACGTC-3′
IL-1β	5′-CCCTGCAGCTGGAGAGTGTGG-3′	5′-TGTGCTCTGCTTGAGAGGTGCT-3
IL-6	5′-AGACAGCCACTCACCTCTTCAG-3′	5′ TTCTGCCAGTGCCTCTTTGCTG-3′,
TNF-α	5′-CTCTTCTGCCTGCTGCACTTTG-3′	5′-ATGGGCTACAGGCTTGTCACTC-3′
iNOS	5′-GCTCTACACCTCCAATGTGACC-3′	5′-CTGCCGAGATTTGAGCCTCATG-3′,
COX-2	5′-CGGTGAAACTCTGGCTAGACAG-3′	5′-GCAAACCGTAGATGCTCAGGGA-3′,
GAPDH	5′-TGACAACTTTGGTATCGTGGAAGG-3′	5′- AGGCAGGGATGATGTTCTGGAGAG − 3′

iNOS: nitric oxide synthetase, COX-2: cyclooxygenase 2

## Measurement of inflammatory cytokines and nitric oxide (NO) by ELISA

The levels of IL-1β (Elabscience, E-EL-H0149), IL-6 (Elabscience, E-EL-H6156), and nitric Oxide (NO) (MyBioSource, MBS8243214) were measured by ELISA according to the manufacturer’s guidelines. Cytokine release was measured in the supernatants of cultured cells post-LPS-priming and GSK-J4 treatment and further compared to non-primed/non-treated (control) cells. The absorbance value was quickly read using the microplate reader at a detection wavelength of 450 nm, as mentioned in the literature (Huang et al. 2020). Results were presented as picograms per milliliter.

## Protein extraction and western blot (WB) technique

RIPA buffer containing phosphatase and protease inhibitors was used to extract the total proteins (Biosharp, China). 10% polyacrylamide gel electrophoresis (PAGE) gels (Bio-Rad) were used to separate a total of 20 µg of proteins, which were then transferred onto PVDF membranes. The membranes were then blocked for one hour at room temperature (RT) using 5% skim milk, and they were then treated with primary antibodies for the entire night at 4 °C. For one hour at room temperature, the membranes were treated with secondary anti-rabbit IgG. Anti-NF-κB p65 and anti-actin were the primary antibodies employed in this investigation. Following the exposure of the protein bands, Gel-pro software was used to assess the findings (Wang et al. 2022).

### Statistical analysis

Statistical analyses were performed using the statistical package of social science software (SPSS) version 20. Quantitative data was presented as mean ± standard deviation (SD). Comparisons among the three independent experimental groups (untreated control, LPS-stimulated, and LPS + GSK-J4-treated cells) were conducted using one-way analysis of variance (ANOVA) followed by an appropriate post hoc multiple-comparison test when data satisfied assumptions of normality and homogeneity of variance, as assessed by the Shapiro–Wilk test and Levene’s test, respectively. For datasets that did not meet parametric assumptions, the non-parametric Kruskal-Wallis test followed by Dunn’s post hoc test with multiplicity adjustment was applied. A two-tailed p-value < 0.05 was considered statistically significant.

## Results

### Study cohort characteristics

Thirty cases were enrolled in the study: 10 RA cases, 10 SLE cases, and 10 patients having IBD (all diagnosed as Crohn’s disease). All participants were adult females and aged between 30 and 55 years, with an average age (49.75 ± 8.65 years for RA, 43.33 ± 7.63 years for SLE, and 32.50 ± 6.61 years for Crohn’s disease cases).

RA cases were clinically evaluated. Family history of RA was positive in one case. Rheumatoid factor (RF) was positive in eight cases. Erythrocyte sedimentation rate (ESR) levels ranged from 19 to 112 mm/h, showing different levels of inflammatory responses. The duration of the disease ranged from 2 to 16 years. The disease activity score in 28 joints (DAS-28) with ESR was 4.27 to 6.06, indicating that our cases had moderate to high disease activity. Joint deformity in the form of flexion deformity of the elbow was identified in six cases. No extra-articular manifestation was observed at the clinical assessments. Concerning treatment, they were all on conventional disease-modifying antirheumatic drugs (DMARDs): methotrexate (20 mg/week), leflunomide (20 mg/day), hydroxychloroquine (200–400 mg/day), and sulfasalazine (Salazopyrin).

Considering the SLE group, the cases showed heterogeneous but characteristic clinical manifestations. All patients were descended from a non-consanguineous marriage, with three of them having a positive family history. All participants presented with classical lupus features, including malar rash (malar flush), inflammatory arthritis, and recurrent chest infections. Only one case showed renal involvement proven on biopsy as lupus nephritis (activity index 2/24; chronicity index 1/22). Cardiovascular involvement was also present in one case as ischemic cardiomyopathy. Overall, these findings are indicative of multisystemic SLE.

In regard to Crohn’s disease cases, two patients had a positive family history, and four patients stated positive consanguinity. Fatigue, diarrhea, vomiting, abdominal pain, bloating, weight loss, and loss of appetite were present in all participants, with nine of them having rectal bleeding. There were extraintestinal manifestations such as osteoporosis (80%), eye irritation (40%), skin rashes (80%), and arthritis (100%). Psychological stress and depression were reported in four patients. Values of fecal calprotectin reflected a high level of intestinal inflammation (112 to 1800 µg/g). Colonoscopy showed fulminant colitis in 9 individuals and chronic ileitis with ulceration in one case correlated with active inflammation.

### Morphological changes of isolated PBMC-derived monocytes and the results of the CCK-8 assay

**Fig. 1:**
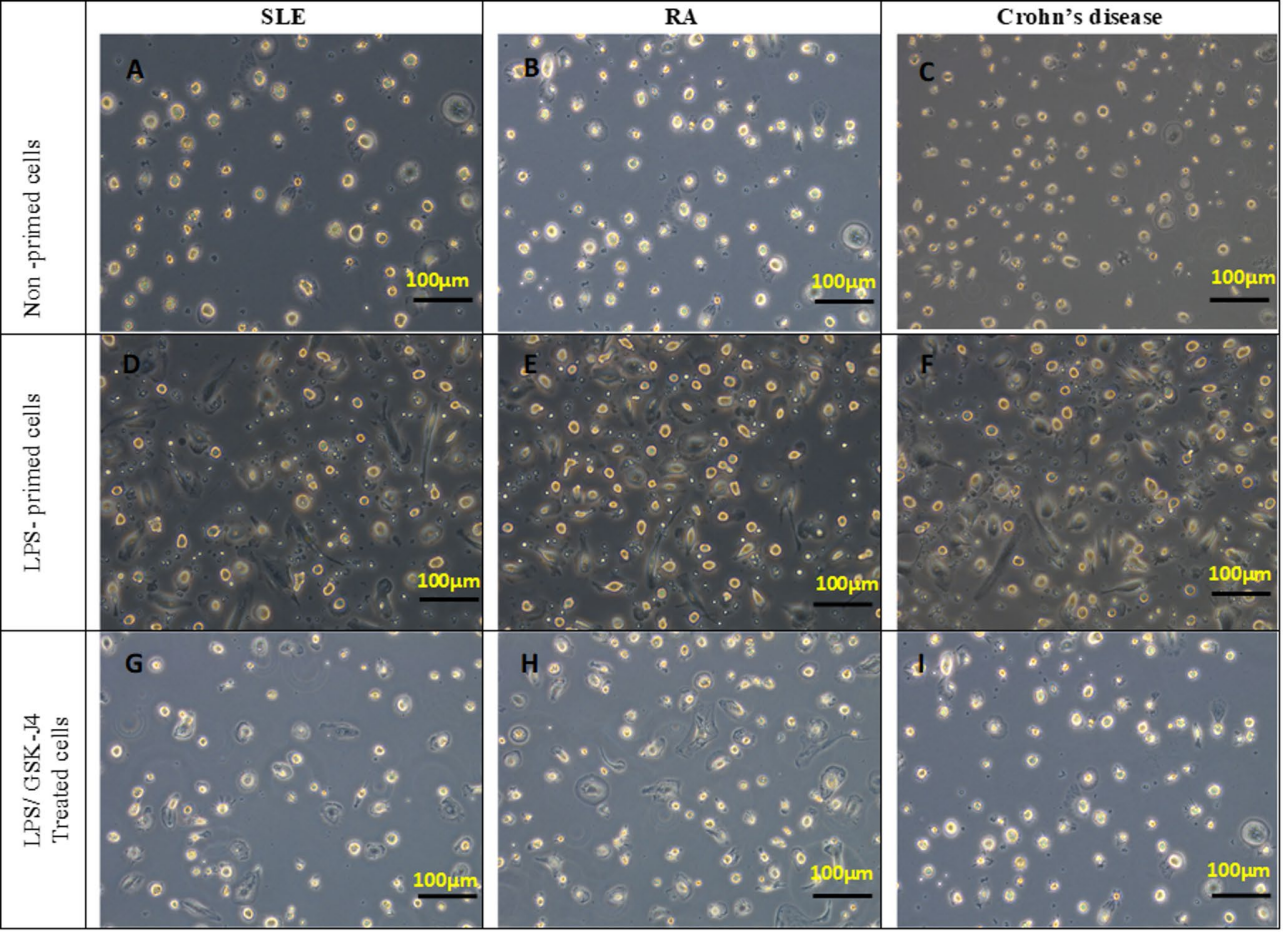
Dynamic morphological changes of PBMCs- deived monocytes isolated from cases with rheumatoid arthritis (RA), systemic lupus erythematosus (SLE), and Crohn’s disease, as depicted in the images, following LPS priming and subsequent GSK-J4 treatment, control cells (**A**–**C**) showed typical morphology, which was rounded and adherent with no signs of membrane blebbing or alteration of the cytoplasmic appearance, indicative of viable and metabolically active immune cells. Conversely, LPS stimulation (**D**–**F**) resulted in significant inflammatory injury, which was characterized by the presence of spindle-shaped cells with condensed nuclei and long pseudopodia on one or both sides of the cells. GSK-J4 treatment (**G**–**I**) significantly reduced the morphological changes associated with LPS stimulation, that were indicative of cellular adherence, reduced cellular debris, and nuclear condensation. All images were taken by an inverted phase contrast microscope with a 20x lens

**Fig. 2:**
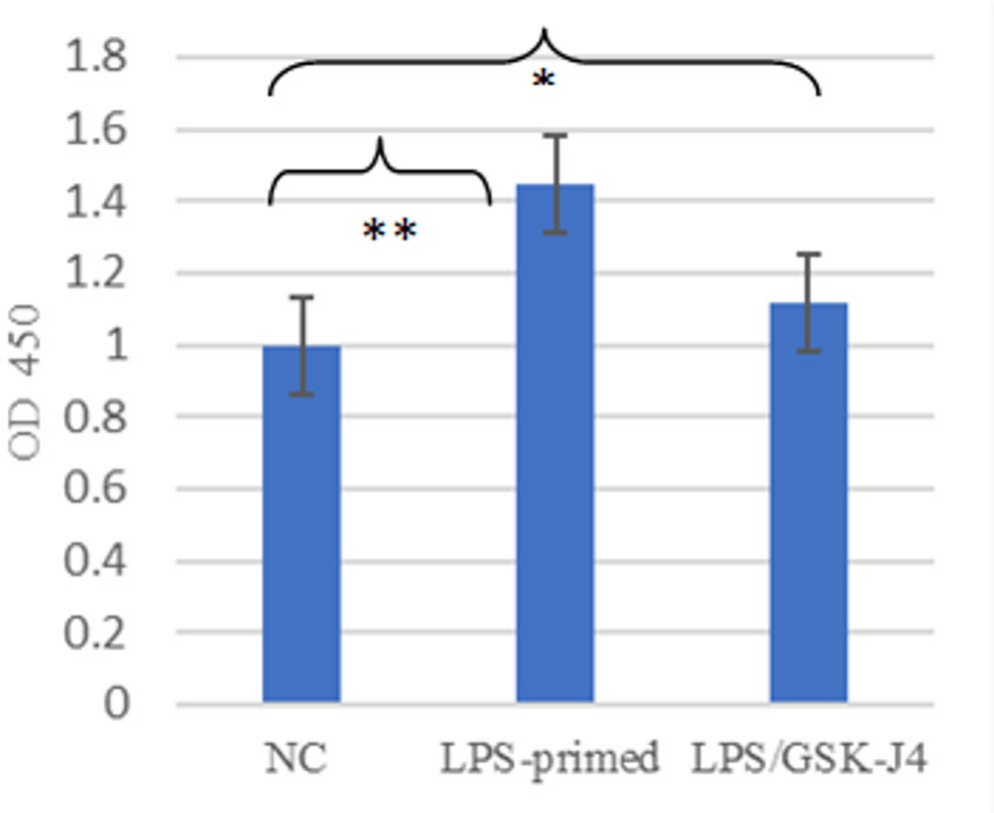
PBMCs proliferation determined by CCK-8 assay for LPS-primed cells and GSK-J4-treated cells in relation to non-primed/non-treated group (NC). *P < 0.05 and **P < 0.01 are considered significant

Prominent morphological changes were detected in both LPS-primed and LPS-induced/GSK-J4-treated cells. The morphological changes for the LPS-primed cells were in the form of a change of the round cells into gathering spindle-shaped cells with extended pseudopodia from one or two sides of the cells (Fig. 1). Following GSK-J4 treatment, morphological changes obviously appeared with a detected decline in cellular proliferation. Nuclear condensation was also observed in the case of LPS-primed cells and was used as a marker of inflammatory cellular stress and chromatin reorganization. The process was attributed to the elevated transcriptional level during inflammasome priming, which is NF-κB dependent. Notably, GSK-J4 treatment was shown to reduce the level of nuclear condensation significantly, thus confirming the role of the compound in the modulation of inflammatory activation. The CCK-8 assay was used to determine the viability of PBMC- derived monocytes. In comparison to the non-primed control cells, LPS priming significantly increased cell proliferation (1.45 ± 0.27, *P* = 0.003). In contrast, the LPS-primed cells following GSK-J4 treatment displayed a substantially lower rate of cellular proliferation (1.12 ± 0.53, *P* = 0.02) compared to the non-treated/non-stimulated control group (Fig. 2).

### Results of JMJD3 inhibition using GSK-J4

#### Differential gene and protein expression following GSK-J4 treatment

To examine the capability of GSK-J4, a small-molecule inhibitor of JMJD3, for impeding NF-κB and subsequent cytokine overproduction, LPS-primed PBMC-derived monocytes from different cell sources were treated with GSK-J4 for 24 h. NF-κB p65 and the tested proinflammatory cytokines, including IL-1β, IL-6, and TNF-α release, were estimated through gene expression profiling. Moreover, the relative quantification of inflammatory mediators, induced nitric oxide synthetase (iNOS), and cyclooxygenase 2 (COX-2) genes was also determined.

**Fig. 3:**
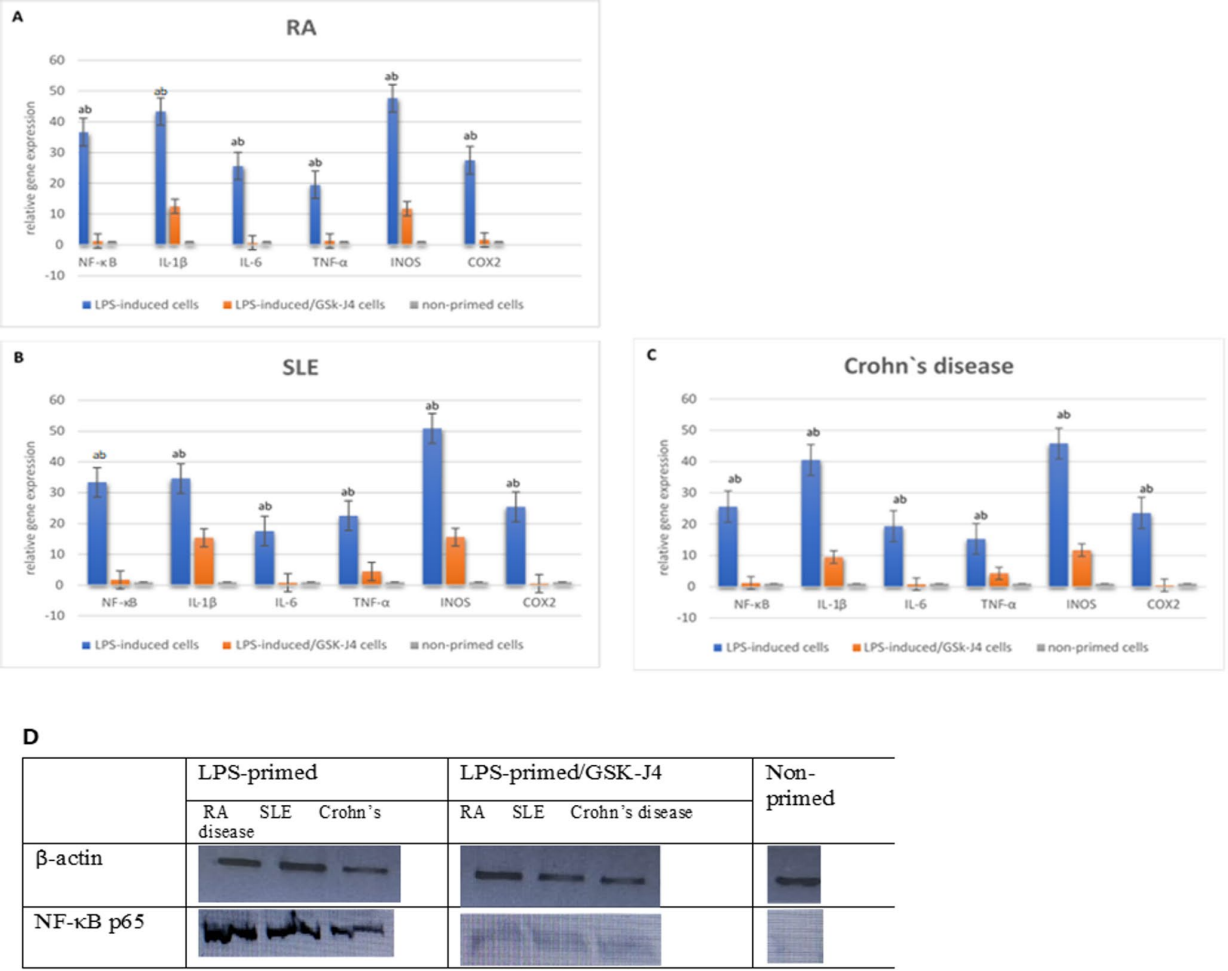
Effect of JMJD3 inhibition by GSK-J4 on inflammatory gene expression in autoimmune disease-derived cells. Relative gene expression of NF-κB p65, IL-1β, IL-6, TNF-α, iNOS, and COX-2 was measured in adherent mononuclear cells derived from patients with **A**: RA, **B**: SLE, and **C**: Crohn’s disease. The cells were measured under three conditions: non-primed control cells, LPS priming of cells, and LPS priming of cells treated with GSK-J4. LPS stimulation was found to induce all the inflammatory markers significantly compared to the non-primed control cells. GSK-J4 was found to inhibit this induction of markers significantly. Statistical symbols: a = significantly different from non-primed control cells; b = significantly different from LPS primed/GSK-J4-treated cells (*p* < 0.05). **D**: Western blot analysis showed that treatment with GSK-J4, following LPS priming, led to a marked decrease in the expression levels of inflammatory protein, NF-κB P65 effectively

In the three groups of cases, mRNA expression of the NF-κB p65 gene and the three cytokines was significantly upregulated in the LPS-induced cells compared to non-induced cells (NC), followed by considerable suppression after GSK-J4 treatment (Fig. 3). Additionally, the relative gene quantification of inflammatory enzymes (iNOS and COX-2) was considerably lower in GSK-J4-treated cells than in primed/non-treated cells (Fig. 3). Comparing the results of gene expression analysis between LPS-induced and LPS/GSK-J4 cells in the three groups of cases is illustrated in Table 2.

Regarding the results of Western blotting (WB) to verify NF-κB protein, all the studied groups exhibited a significant depression of NF-κB in LPS-induced cells after GSK-J4 treatment in relation to non-treated cells, which was consistent with the results of gene expression analysis (Fig. 3).

**Table 2:** The results of gene expression analysis in LPS-induced cells from different autoimmune diseases before and after GSK-J4 treatment

Gene	RA			SLE			Crohn’s disease		
	LPS-induced cells	LPS-induced/GSK-J4 cells	*P*-value	LPS-induced cells	LPS-induced/GSK-J4 cells	*P*-value	LPS-induced cells	LPS-induced/GSk-J4 cells	*P*-value
NF-κB p65	36.65 ± 2.86	1.26 ± 0.15	0.008*	33.35 ± 5.16	1.71 ± 0.37	0.003*	25.61 ± 2.35	1.2 ± 0.29	0.003*
IL-1β	43.35 ± 2.98	12.54 ± 1.14	0.001*	34.52 ± 2.22	15.45 ± 0.82	0.001*	40.53 ± 2.04	9.48 ± 0.81	< 0.001*
IL-6	25.64 ± 2.70	0.72 ± 0.21	0.004*	17.59 ± 2.19	0.77 ± 0.15	0.005*	19.38 ± 1.49	0.78 ± 0.14	0.002*
TNF-α	19.58 ± 3.47	1.33 ± 0.19	0.011	22.57 ± 1.67	4.45 ± 0.47	0.002*	15.33 ± 1.83	4.28 ± 0.38	0.007*
iNOS	47.63 ± 2.96	11.82 ± 1.68	< 0.001*	50.81± 3.249	15.61 ± 0.61	0.002*	45.78 ± 2.29	11.72 ± 1.65	< 0.001*
COX-2	27.52 ± 3.21	1.65 ± 0.41	0.005*	25.41 ± 2.79	0.56 ± 0.19	0.004*	23.55 ± 1.85	0.43 ± 0.10	0.002*

The gene expression findings were presented as Mean ± standard deviation (SD), *P ≤ 0.05 is considered significant.

### Measurement of proinflammatory cytokines and oxidative stress marker (NO) by ELISA technique

**Fig. 4:**
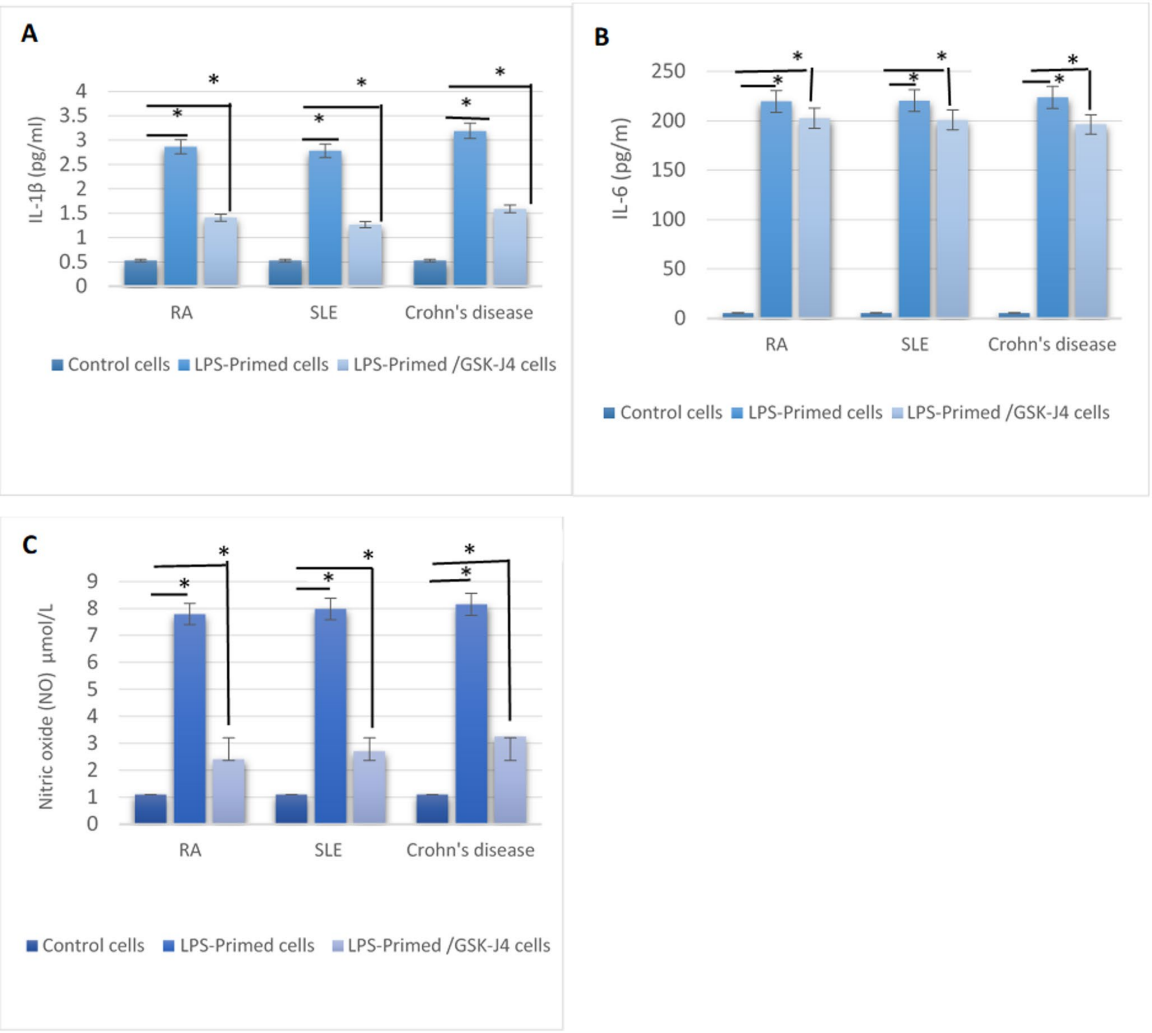
GSK-J4 suppressed LPS-induced inflammatory mediator release in primary immune cells from autoimmune disease patients. **A**–**C** ELISA quantification of IL-1β (**A**), IL-6 (**B**), and NO (**C**) in patient-derived adherent immune cells from RA, SLE, and Crohn’s disease under three experimental conditions: untreated control cells, LPS-primed cells, and LPS-primed / GSK-J4-treated cells. LPS stimulation significantly increased cytokine and NO production compared with untreated control cells in all disease groups. Treatment with GSK-J4 following LPS priming significantly reduced IL-1β, IL-6, and NO levels relative to LPS-stimulated cells, although values remained above baseline control levels. Data are presented as mean ± SD. Statistical significance was determined as *p < 0.01. Lines indicate pairwise comparisons: control vs. LPS-primed cells and LPS-primed vs. LPS/GSK-J4-treated cells

Significant release of IL-1β and IL-6 was confirmed by ELISA. Regarding LPS-primed cells, IL-1β recorded 2.86 ± 0.29, 2.78 ± 0.38, and 3.19 ± 0.41 pg/ml, while IL-6 recorded 219.67 ± 4.51, 220.33 ± 3.21, and 223 ± 4.12 pg/ml in RA, SLE, and Crohn’s disease, respectively, with significant variation in relation to non-primed/non-treated control cells (NC) (Fig. 4). On GSK-J4 treatment, IL-1β was reduced to 1.41 ± 0.27, 1.27 ± 0.21, and 1.62 ± 0.4 pg/ml, while IL-6 recorded 202.67 ± 2.5, 200.83 ± 4.54, and 196 ± 7.02 pg/ml in RA, SLE, and Crohn’s disease, respectively, with P-value reaching the significant level compared to the pretreatment levels (Fig. 4). The inflammatory mediator (NO) release was also estimated for the three groups of patients, and it was significantly higher in LPS-induced cells (7.8 ± 0.82, 7.99 ± 0.58, and 8.16 ± 0.82 µmol/L) than in LPS/GSK-J4 cells (2.41 ± 0.52, 2.7 ± 0.49, and 3.24 ± 0.24 µmol/L) in RA, SLE, and Crohn’s disease (Fig. 4).

## Discussion

Autoimmune diseases (AIDs) emerge from irregular immune responses against self- and non-self-antigens, with inflammatory factors and inflammasomes playing central roles in their progression. Prior reports have documented that deregulated inflammasome activation is responsible for the development of autoimmune/autoinflammatory, metabolic diseases, neurodegenerative disorders, and cancer (Raneros et al. 2021). Autoimmune diseases such as RA, IBD and SLE have autoimmune and inflammatory backgrounds of illness in which NF-κB contributes to immune activation, including innate cells and adaptive cells, besides pro-inflammatory cytokine release, causing more aggravation of disease pathogenesis (Xie et al. 2019; Zhang et al. 2021; and Pang et al. 2023). DNA methylation and histone modifications play a key role in modulating the expression of inflammasome components. Thereby, focusing on treatment strategies directed towards inflammatory mediators, pathways, and inflammasome deregulation presents a promising strategy for managing AIDS (Xiang et al. 2023).

JMDJ3 is one of the epigenetic players that could be rapidly induced by NF-κB. According to Wei et al. (2013), JMJD3 has also been known to boost NF-κB, creating a positive feedback loop and promoting its transcription. The promoter area of JMJD3 consists of two conserved kB sites, suggesting its association with NF-kB signaling. Moreover, NF-kB has bonding sites on the promotor of iNOS and COX-2 genes; thereby, NF-kB stimulation and expression during inflammation enhance their expression with remarkable production of NO and other inflammatory prostaglandins (Adel et al. 2024).

Meanwhile, GSK-J4 remains the most effective and potent tool for blocking the JMJD3 demethylase activity. It has been described to limit inflammation with declined levels of proinflammatory cytokines (Pan et al. 2018) and more recently reported to affect T-cell differentiation in vitro (Liu et al. 2015).

Therefore, our goal was to elucidate the effect of JMJD3 inhibition by its inhibitor, GSK-J4, on inflammasome activation and oxidative stress release in LPS-stimulated cells from cases having different autoimmune diseases: RA, IBD, and SLE. Accordingly, the patients’ PBMC-derived monocytes, were isolated and exposed to LPS, which is one of the typical stimulating molecules of NF-κB (Schmid et al. 2008). Subsequently, GSK-J4 was applied to determine its ability to alter the adherent PBMC-induced inflammatory response. LPS-primed cells across the three groups of patients showed a change in the morphological characteristics that was further altered upon GSK-J4 treatment, with a discernible decrease in nuclear condensation and cellular proliferation. Moreover, LPS-priming significantly boosted cell proliferation compared to the non-stimulated (control) cells (all *P* < 0.01). However, LPS/GSK-J4-treated cells showed a significantly lower rate of cellular proliferation in relation to the control cells.

Furthermore, the gene expression of NF-κB p65 and proinflammatory cytokines (IL-1β, IL-6, and TNF-α) was monitored in LPS-primed cells from the three AIDs before and after GSK-J4 treatment, and the GSK-J4-treated cells exhibited significantly reduced expression of NF-κB p65 and the proinflammatory cytokines in comparison to primed/non-treated cells (all p **≤** 0.01). Additionally, relative gene quantification of the inflammatory enzymes (iNOS and COX-2) was also estimated and showed substantial downregulation in LPS-induced cells after treatment with GSK-J4. From Western blot analysis, the expression of NF-κB was highly suppressed following GSK-J4 treatment in accordance with gene expression results of NF-κB.

ELISA results in our experiment further disclosed a considerable release of IL-1β and IL-6 alongside NO in the stimulated cells that was significantly reduced following GSK-J4 application.

Our results were concomitant with previously reported studies that have described the ability of GSK-J4 to alleviate LPS-induced in vitro synthesis of pro-inflammatory cytokines in macrophages (Kruidenier et al. 2012; Lu et al. 2023). Similarly, it has been described by Ding and colleagues (2023), that JMJD3 blockage attenuates the migration and proliferation of fibroblast-like synoviocytes (FLS), the key player in the pathological course of RA. Zhao’s research. (2022b) also provided evidence that GSK-J4 could improve the RA manifestations in Collagen-Induced Arthritis (CIA) mice by repressing the expression of IL-6 and other inflammatory cytokines in macrophages through enhancing the level of H3K27me3. Additionally, a previous study also documented that GSK-J4 could suppress IL-1β-stimulated NF-κB signaling in vitro, prevent IL-1β-induced collagen II breakdown, and further stop the cartilage degeneration in an in vivo model of osteoarthritis (Jun et al. 2020).

Regarding SLE, Mei and co-authors (2023) reported that JMJD3 participates in regulating CD11a expression in lupus T cells by changing histone H3K27 tri-methylation levels, promoting T-cell proliferation in SLE cases, and accentuating disease phenotype. They further suggested the role of targeted inhibition of JMJD3 in improving the disease progression in SLE individuals. Moreover, the effects of GSK-J4 on T-cell differentiation in vitro were previously addressed by Liu et al. (2015), who recorded that JMJD3-deficient mice were resistant to the induction of experimental autoimmune encephalomyelitis (EAE). Similarly, Doñas et al. (2016) also observed that giving GSK-J4 to EAE mouse models delayed disease onset and led to milder symptoms, suggesting its protective effect on autoimmune disease.

Finally, the effect of JMJD3 in the advancement of immune colitis has been previously explored by Huang and colleagues (2020), who found that the JMJD3 inhibitor, GSK-J4, greatly downregulated NLRP3 inflammasome assembly through enhancing the recruitment of H3K27me3 to the Nrf2 promoter, hindering Nrf2 activation. They also indicated the crucial function of JMJD3 as a future target for IBD treatment.

Therefore, GSK-J4 has a potent anti-inflammatory activity in RA, SLE, and Crohn’s disease in the context of a common epigenetic mechanism in the three examined diseases. It seems that epigenetic regulators such as JMJD3 represent potential targets to regulate the inflammatory response in several autoimmune diseases (Allis et al. 2016; Leng et al. 2022).

This study demonstrated a novel aspect of epigenetic regulation in autoimmune inflammation since it is the first time that three autoimmune diseases have been analyzed using one experimental model. It employed patient-derived primary immune cells, which increased the relevance of the findings. The integrated validation strategy incorporated gene expression profiling, protein analysis, cytokine quantification, and morphologic assessment. The results suggested that JMJD3-dependent NF-κB signaling epigenetic regulation represents a pathogenic mechanism commonly shared by a group of autoimmune diseases, thus revealing a shared therapeutic target. Ultimately, this study had a few drawbacks that need to be mentioned. In this regard, immunophenotypic characterization should have been conducted, although the monocyte-derived cells formed a majority of the adherent cell population that was investigated (Arteche-Villasol et al. 2020). In addition, the in vitro experimental framework is not sufficient to represent the complexity of tissue-specific microenvironments and in vivo immune interactions. Future studies that integrate dose-response, in vivo validation, and epigenetic mapping are expected to reveal the therapeutic potential of suppressing JMJD3.

## Conclusion

The present investigation demonstrated that pharmacological inhibition of JMJD3 effectively suppresses the inflammatory pathways controlled by NF-κB in immune cells from patients with varied autoimmune diseases. These findings also suggested the potential of histone demethylases as prospective therapeutic targets for immune-mediated chronic inflammatory diseases and emphasized the key role of epigenetics in modulating autoimmune diseases.

## Data Availability

All data generated or analyzed during the study is included in the manuscript.
